# What we already know about rhubarb: a comprehensive review

**DOI:** 10.1186/s13020-020-00370-6

**Published:** 2020-08-26

**Authors:** Hong Xiang, Jiaxin Zuo, Fangyue Guo, Deshi Dong

**Affiliations:** 1 Laboratory of Integrative Medicine, First Affiliated Hospital of Dalian Medical University, Dalian, China; 2 College of Pharmacy, Dalian Medical University, Dalian, China; 3 Institute (College) of Integrative Medicine, Dalian Medical University, Dalian, China; 4 Department of Clinical Pharmacy, First Affiliated Hospital of Dalian Medical University, Dalian, China

**Keywords:** Rhubarb, TCMs, Taxonomic identification, Pharmacology, Clinical application

## Abstract

Rhubarb (also named Rhei or Dahuang), one of the most ancient and important herbs in traditional Chinese medicine (TCM), belongs to the *Rheum L.* genus from the Polygonaceae family, and its application can be traced back to 270 BC in “*Shen Nong Ben Cao Jing*”. Rhubarb has long been used as an antibacterial, anti-inflammatory, anti-fibrotic and anticancer medicine in China. However, for a variety of reasons, such as origin, variety and processing methods, there are differences in the effective components of rhubarb, which eventually lead to decreased quality and poor efficacy. Additionally, although some papers have reviewed the relationship between the active ingredients of rhubarb and pharmacologic actions, most studies have concentrated on one or several aspects, although there has been great progress in rhubarb research in recent years. Therefore, this review aims to summarize recent studies on the geographic distribution, taxonomic identification, pharmacology, clinical applications and safety issues related to rhubarb and provide insights into the further development and application of rhubarb in the future.

## Background

Rhubarb is one of the most ancient and important herbs with thick roots, hollow and erect stems and small white-green or purple-red flowers clustered on the branches [1]. Rhubarb includes approximately 60 species of plants of the genus *Rheum L*. from the Polygonaceae family [2]. The rhizome of rhubarb was classified as a top medicinal plant, which can be traced back to 270 BC in an ancient Chinese book “*Shen Nong Ben Cao Jing*” [3]. Rhubarb has mainly been used for medicinal purposes in Asia, but it often refers to a few edible rhubarbs in Europe and the Middle East. Modern studies of rhubarb have identified the chemical constituents [4], pharmacological activities [5, 6] and functional mechanisms [7] in a more scientific and rigorous way. Although some papers have reviewed the relationships between the active ingredients of rhubarb and pharmacologic actions [2], most of these studies have concentrated on one or several aspects, although research on rhubarb has made great progress in recent years. In this review, we not only summarize the leading-edge understanding in certification methods, quality control, pharmacology and clinical applications of rhubarb but also introduce the geographic distribution, taxonomic identification and toxicity. This review delivers multifaceted and different views and opinions from the field, which will provide insights into the application of rhubarb and be helpful for increasing awareness of the diversity and situations in which the biological resources of rhubarb plants are used.

## Global geographic distribution of rhubarb

**Fig. 1:**
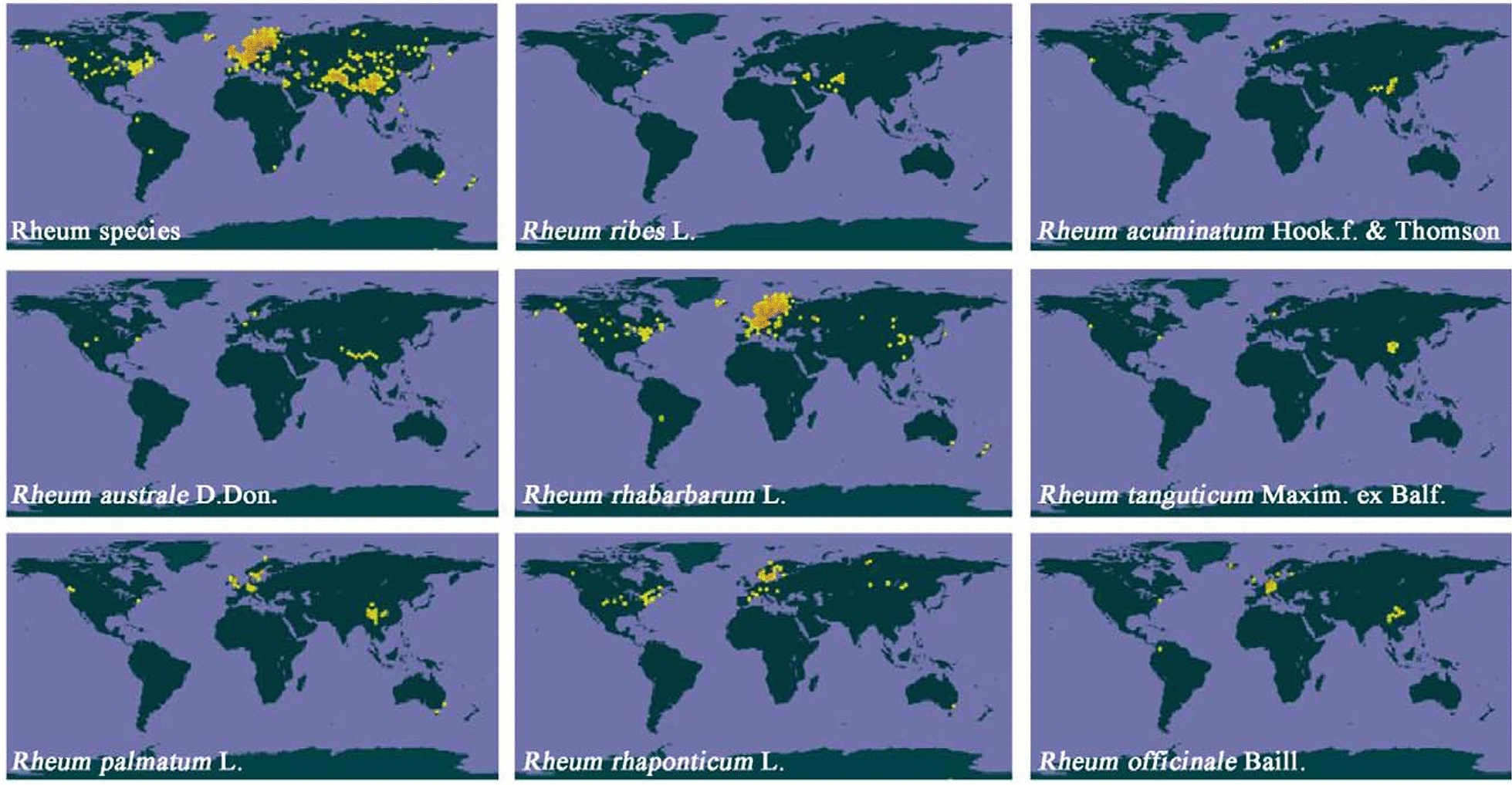
Geographical distribution of Rhubarb species. Data from the Global Biodiversity Information Facility (https://www.gbif.org/)

According to the database of the Global Biodiversity Information Facility (https://www.gbif.org/), 23 rhubarb species are recorded. As shown in Fig. 1, rhubarb species including *R. tanguticum* Maxim., *R. officinale* Baill., *R. palmatum* L., *R. acuminatum* Hook. f. & Thomson., *R. australe* D. Don. are mainly distributed in Europe and the southwestern area of China, while *R. rhabarbarum* L. and *R. rhaponticum* L. are distributed widely across Europe to North America and part of Asia, showing obviously different geographical distributions. As the distribution center of rhubarb, there are 39 species and 2 varieties in China, most of which are concentrated in northwest and southwest China. *R. palmatum* L. grows in forest margins in mountainous regions of China, such as in the provinces of Sichuan, Gansu, Qinghai and Tibet, and *R. tanguticum* Maxim. and *R. officinale* Baill. grow in well-drained mountainous areas, such as in Hubei, Sichuan, Yunnan and Guizhou Provinces [8]. Three species of Rheum, including *R. tanguticum* Maxim., *R. officinale* Baill. and *R. palmatum* L. have been officially adopted into both the *Chinese Pharmacopoeia* and *Korean Pharmacopoeia* using the common drug name “Dahuang”. *Japanese Pharmacopoeia* prescribes the three species together with *R. coreanum* and their interspecific hybrids as origins for medicinal rhubarb [9].

## Taxonomic identification

### Traditional and classic methods

The quality of traditional Chinese medicine (TCM) is closely related to its variety and origin. With the expansion of market demand, rhubarb has been planted in a larger range of areas. Several species similar to rhubarb (such as *Rumex crispus*, *Rumex aquatica* and *Reynoutria elliptica*) are frequently used in medicinal formulations and labeled as rhubarb [9], which often confuses researchers and doctors, and quality degradation that affects therapeutic efficacy has also been pointed out by doctors [8]. Accordingly, much effort has been focused on the taxonomic identification of these various species, and some progress has been made. Traditional methods used to identify rhubarb are mainly based on the characteristics of the flowers, leaves, cross-sections of roots, and crystal characteristics of the powder. The characteristics of the leaves and flowers can be used to identify different species of rhubarb, which was recorded in “*Zhi Wu Ming Shi Tu Kao*”, a Chinese botanical work initially published in 1848, and rhubarb morphology, as the first step of screening, is still widely used in the field detection of species. Rhubarb listed in the Pharmacopoeia comes from the root and stem of *R. tanguticum* Maxim., *R. officinale* Baill. and *R. palmatum* L., while other rhubarb species, including *R. franzenbachii* Münter., *R. australe* D. Don., *R. nobile* Hook.f. & Thomson. and *R. wittrockii* C.E. Lundstr., are often misused in the treatment of diseases with weak efficacy. Therefore, we describe the characteristic morphology of the above seven rhubarb species in Table 1, which can provide some information for the identification of rhubarb [10]. Briefly, all three kinds of medicinal rhubarb have leaf splits with obviously different depths and shapes, and the other four species of rhubarb have no splits on their leaves with lower heights.

**Table 1:** Characteristic morphologies of seven rhubarb species

Species	Height	Flowers	Leaves shapes	Leaves splits
*R. tanguticum* Maxim.	1.5–2 m	Small, purple or light red	Subcircular or broadly ovate, 30–60 cm long, narrow and pointed apex	About 5 splits, needle-shaped, closely to the base of the leaf
*R. officinale* Baill.	1.5–2 m	Large, yellowish-white	Subcircular, 30-50 cm in diameter, pointed apex	Shallow, only 1/5 to 1/4, triangular shape
*R. palmatum* L.	1.5–2 m	Small, purple-red or yellow-white	Subcircular, 40–60 cm in diameter, pointed apex	About 1/3 to 1/4, triangular shape
*R. franzenbachii* Münter.	0.5–0.9 m	Yellow-white, 3-6 clusters	Cardiac ovate, 12–22 cm long, 10–18 cm wide, corrugated margin	Few or no
*R. australe* D. Don.	0.7–2 m	Purple-red	Oval-elliptic, 20–50 cm long, 18–40 cm wide, margin with weak wrinkles	Few or no
*R. nobile* Hook.f. & Thomson.	1–2 m	Yellow-green, 5–9 clusters	Lotus-shaped, 20–30 cm in diameter, round apex	Few or no
*R. wittrockii* C.E. Lundstr.	0.5–1 m	White-green, small, 2 mm in diameter	Ovate, 15–26 cm long, 10–20 cm wide, obtuse and acute apex, margin with weak wrinkles	Few or no

However, it is difficult to correctly identify the exact species due to the similarity between species and morphological variation in the leaves. Therefore, the 10th China Pharmacopoeia Committee introduced and stated three methods to identify rhubarb: 1) the first two methods are based on their root cross-sectional characteristics and powder crystal morphology; 2) the third method is thin-layer chromatography (TLC), which requires five major orange fluorescent spots on the chromatographic phase at the same location as the control, and exposure to ammonia vapor turns the orange to red.

### Modern identification methods

The information provided by the traditional qualitative methods mentioned above is too little to distinguish plant species with similar appearances or chemical compositions [11]. Rhubarb can be used to treat multiple diseases, which is related to the complex chemical composition caused by plant species, growth environment, harvest time, processing and so on [8]. Therefore, methods that can identify the most components in rhubarb are widely used for species identification and quality identification (Table 2). The major constituents of rhubarb are a variety of phenolic compounds, such as anthraquinone derivatives, dianthrones, stilbenes, polyphenols, flavonoids and chromones. Among them, rhein, emodin, aloe-emodin, physcion and chrysophanol are well recognized as biologically active components. Their contents are often used as a criterion for the quality control of rhubarb products. Various methods have been proposed for the quantitative determination of the five major anthraquinones based on high-performance liquid chromatography (HPLC), capillary zone electrophoresis (CZE), micellar electrokinetic chromatography (MEC), TLC and many other methods. Each method has its own advantages, but no summary and comparison exist at present, which is not conducive to the selection of content determination methods. HPLC remains the mainstream analytical method for rhubarb. VanMen et al. developed an HPLC-based method and successfully selected 17 peaks with rhubarb, including 5 pharmacologically active compounds. Ninety-six samples were separated into 5 species based on linear discriminant analysis with an accuracy of 100% [9]. However, using HPLC alone either provides poor sensitivity or is time consuming due to the variations in rhubarb species and concentrations of these compounds in samples [12]. To improve sensitivity and increase the analysis efficiency, capillary HPLC has been used in quality control analyses of rhubarb [13]; additionally, HPLC coupled with many detectors, such as ultraviolet (UV) [14], diode array detector (DAD) [4], capillary electrophoresis (CE) [15], and mass spectrum (MS) [16], has been developed, which makes it more possible to detect the different components [11]. The fingerprint method for the systematic study of the chemical constituents of TCMs can be used to indicate the chemical characteristics of TCMs by chromatogram or spectrogram. This method has been internationally applied to evaluate the authenticity, goodness and stability of TCMs. Jin et al. successfully established a fingerprint method using high-performance liquid chromatography- photodiode array detection (HPLC–DAD) to control the quality of *R. tanguticum* [4]. This HPLC fingerprint analysis method provides an important reference for the establishment of quality control methods for other rhubarb varieties.

**Table 2:** Quality control and chemical analysis of rhubarb extracts

No.	Extracts	Species	Part	Method	Quality control	Refs.
1	50% ethanol extract	*R. palmatum* L.	Root	Column chromatography; HPLC	Aloe-emodin, rhein, emodin, chrysophanol, physcion	[107]
2	–	*R. palmatum* L.	Rhizome	–	Aloe-emodin (0.11%), rhein (0.37%), emodin (0.05%), chrysophanol (0.36%), physcion (0.26%)	[14]
3	Methanol extract	*R. palmatum* L. (Three-year-old; harvested in September)	Root	HPLC	Aloe-emodin (0.21%), rhein (0.21%), emodin (0.37%), chrysophanol (1.09%), physcion (0.24%)	[108]
4	Methanol extract	*R. palmatum* L. (Three-year-old; harvested in September)	Root	^1^H-NMR	Aloe-emodin (0.23%), rhein (0.19%), emodin (0.38%), chrysophanol (1.26%), physcion (0.23%)	[108]
5	–	*R. tanguticum* Maxim.	Rhizome	–	Aloe-emodin (2.09%), rhein (0.001%), emodin (0.001%), chrysophanol (0.08%), physcion (0.07%)	[14]
6	–	*R. officinale* Baill.	Rhizome	–	Rhein (0.001%), chrysophanol (0.09%), physcion (0.09%)	[14]
7	Methanol extract	–	Root	RP-HPLC	Aloe-emodin, rhein, emodin, chrysophanol, physcion	[109]
8	Chloroform extract	–	–	MEEKC	Aloe-emodin (2.04%), rhein (0.5%), emodin (1.58%), chrysophanol (5.06%), physcion (0.98%)	[110]
9	95% ethanol and chloroform extract	–	–	DryLab software-aided RP-HPLC	Aloe-emodin (0.15%), rhein (0.03%), emodin (0.09%), chrysophanol (0.52%), physcion (0.05%)	[111]
10	Chloroform extract	–	–	DryLab software-aided RP-HPLC	Aloe-emodin (0.33%), rhein (0.07%), emodin (0.20%), chrysophanol (1.02%), physcion (0.18%)	[111]
11	Water extract	–	–	UPLC/Q-TOF-MS/MS	Aloe-emodin (0.14%), rhein (0.31%), emodin (0.24%), chrysophanol (0.37%), physcion (2.69%)	[112]
12	Ethanol-chloroform extract	–	–	LC–MS	Rhein, physcion, emodin	[113]
13	Methanol extract	–	–	BC-SOS-LPME coupled with HPLC–UV	Rhein (0.6%), chrysophanol (2.53%)	[114]
14	Methanol extract	*Rhei Rhizoma*	–	HPLC–UV-MS	Aloe-emodin (0.24%), rhein (0.13%), emodin (0.11%), chrysophanol (0.31%), physcion (0.13%)	[115]
15	80% methanol extract	*R. officinale*; *R. palmatum;* *R. Tanguticum*	–	UPLC-PDA	Aloe-emodin-8-O-glucoside, rhein-8-O-glucoside, chrysophanol-1-O-glucoside, emodin-1-O-glucoside, chrysophanol-8-O-glucoside, emodin-8-O-glucoside	[116]
16	Methanol extract	*R. undulatum* L.	Rhizome	Diaion HP-20 column chromatography; silica gel column chromatography; ODS column chromatography; HPLC	Chrysophanol-8-O-β-d-(6′-galloyl)-glucopyranoside, aloe-emodin-1-O-β-d-glucopyranoside, chrysophanol-1-O-β-d-glucopyranoside, chrysophanol-8-O-β-d-glucopyranoside	[117]
17	Ethanol extract	Korean rhubarb	Rhizome	Silica gel column chromatography	Chrysophanol, physcion, emodin, chrysophanol-8-O-β-d-glucopyranoside, emodin-8-O-β-d-glucopyranoside	[118]
18	Ethanol extract	Korean rhubarb	Rhizome	Silica gel column chromatography	Chrysophanol-8-O-β-d-glucopyranoside, chrysophanol	[119]
19	70% ethanol extract	*R. crispus*	Rhizome	HPLC	Emodin-glucoside (0.006%), emodin (0.12%), chrysophanol (0.39%)	[9]
20	50% ethanol extract	*R. likiangense* Sam.	Rhizome	HSCCC	Rhaponticin, desoxyrhaponticin	[120]
21	Ethanol extract	–	Rhizome	Silica gel column chromatography	Piceatannol-3′-O-β-d-glucopyranoside	[121]
22	Ethanol extract	Korean rhubarb	Rhizome	Silica gel column chromatography	Desoxyrhaponticin, rhaponticin, resveratrol, desoxyrhapotigenin, rhapontigenin, piceid, ε-viniferin, piceatannol-3′-O-β-d-glucopyranoside, ampelopsin B, isorhaponticin	[118]
23	Acetone extract	Garden rhubarb (hybrids of *R.* *rhabarbarum L. and R. rhaponticum L.)*	Rhizome	MLCCC; HPLC; NMR; HR-MS; HPLC–DAD	Trans-rhapontigenin, trans-desoxyrhapontigenin, trans-rhaponticin, trans-desoxyrhapontici, trans-piceatannol-3′-O-β-d-glucopyranoside, trans-resveratrol-4′-O-β-d-glucopyranoside	[122]
24	Methanol extract	*Rhei Rhizoma*	–	HPLC–UV-MS	Resveratrol-4’-O-β-d-glucopyranoside, resveratrol-4′-O-β-d-(2′’-O-galloyl)-glucopyranoside, resveratrol 4’-O-β-d-(6’’-O-galloyl)-glucopyranoside	[115]
25	80% ethanol extract	*R. undulatum* L.	Rhizome	Chromatography	Desoxyrhapontigenin	[123]
26	Methanol extract	*R. undulatum* L.	Rhizome	–	Rhaponticin, rhaponticin 2’’-O-gallate, isorhapontin, piceatannol 3’-O-Glc, rhapontigenin, resveratrol, isorhapontigenin, piceatannol, desoxyrhapontigenin	[124]
27	Methanol extract	*Rhei Rhizoma*	–	–	Rhubarb galloyl (RG)-tannin	[125]
28	Methanol extract	*R. tanguticum* Maxim.	Rhizome	Reversed phase gel column chromatography	RG-Tannin (3.15%), rhatannin (0.005%)	[14]
29	Methanol extract	*R. palmatum* L.	Rhizome	Reversed phase gel column chromatography	RG-Tannin (3.49%), rhatannin (0.85%)	[14]
30	Methanol extract	*R. officinale* Baill.	Rhizome	Reversed phase gel column chromatography	RG-Tannin (1.74%), rhatannin (0.004%)	[14]
31	Methanol/ chloroform extract	*R. emodi*	Rhizom; root	Column chromatography; TLC	–	[126]
32	Methanol extract	*rhaponticum* L.	Petiole	RP-HPLC–UV-ESI/MS^2^	Myricetin-O-rhamnoside, quercetin-O-rutinoside (rutin), quercetin-O-glucoside, quercetin-O-glucuronide, quercetin-O-glucuronide, quercetin	[127]
33	Acetone extract	Garden rhubarb (hybrids of *R.* *rhabarbarum L. and R. rhaponticum L.)*	Leaves; petiole	MLCCC; HPLC; NMR; HR-MS; HPLC–DAD	6,8-di-C-β-d-glucosylapigenin, 6-C-β-d-glucosyl-8-C-β-d-arabinosylapigenin, rutin, 6-C-β-d-arabinosyl-8-C-β-d-glucosylapigenin, quercetin-3-O-β-d-glucuronide, isovitexin	[122]
34	Methanol extract	*Rhei Rhizoma*	–	HPLC–UV-MS	Sennidin A (1.12%), sennidin B (0.37%)	[115]
35	–	Hokkai Daio	Root	Eastern blotting	Sennidin A phloem (0.00584%), radiate wood (0.0049%), pith (0.00133%)	[128]
36	–	*R. australe* D. Don	Root	Column chromatography	Carpusin, maesopsin	[129]
37	Methanol extract	*R. undulatum* L.	Rhizome	Diaion HP-20 column chromatography; silica gel column chromatography; ODS column chromatography; HPLC	Torachrysone 8-O-β-d-glucopyranoside	[117]
38	n-pentane and diethyl ether extract	*R. rhabarbarum* L.	Stalk	VHS; GC–MS; GC	Alcohols, aldehydes, esters, ketones, acids sesquiterpenes, terpenes, phenolic derivatives	[130]
39	Methanol extract	*nanum; R. Racemiferum;* *delavay;* *pumilum; R. sublanceolatum*	–	HPLC–DAD-ESI-MSn	Sennosides, anthraquinones, stilbenes, glucose gallates, naphthalenes, procyanidins, chromones	[131]
40	Methanol extract	*Rhei Rhizoma*	–	HPLC–UV-MS	Gallic acid (0.26%)	[115]
41	95% ethanol extract	–	Root; stem	CCC; LC/MS	Tannins and gallic acid	[132]
42	Ethanol extract	*R. palmatum* L.	Rhizom; root	HPLC	–	[133]
43	Methanol extract	*R. undulatum* L.	Rhizome	Silica gel column chromatography	5-methoxy-cis-rhapontigenin, 5-methoxy-trans-rhapontigenin, resveratrol, piceatannol, deoxyrhapontigenin, rhapontigenin, piceatannol, resveratroloside, δ-viniferin	[134]
44	Methanol extract	*R. emodi* Wall.ex Meisson	Rhizome	Silica gel column chromatography	Desoxyrhapontigenin, desoxyrhaponticin, chrysophanol-8-O–β-d-glucopyranoside, rhapontigenin, torachrysone-8-O-β-d-glucopyranoside	[135]
45	Methanol extract	*R. rhaponticum* L.	Root	RP-HPLC- UV-ESI/MS^2^	Piceatannol glucoside-1, piceatannol glucoside-2, resveratrol glucoside-1, piceatannol glucoside-3, rhapontin-1, resveratrol galloylglucoside, emodin, rhapontigenin, galloylglucoside, deoxyrhapontin, torachrysone glucoside, emodin glucoside, chrysophanol glucoside, deoxyrhapontigenin galloylglucoside, resveratrol dimer-1, resveratrol dimer-2	[127]
46	70% ethanol extract	*R. officinale* Baill.	Rhizome	HPLC	Sennoside A (0.33%), emodin-glucoside (0.04%), chrysophanol (0.21%), emodin (0.03%)	[9]
47	70% ethanol extract	*R. palmatum* L.	Rhizome	HPLC	Sennoside A (0.15%), emodin-glucoside (0.03%), chrysophanol (0.63%), emodin (0.19%)	[9]
48	70% ethanol extract	*R. tanguticum* Maxim.	Rhizome	HPLC	Sennoside A (0.56%), emodin-glucoside (0.05%), chrysophanol (0.38%), emodin (0.11%)	[9]
49	70% ethanol extract	*R. undulatum* L.	Rhizome	HPLC	Rhaponticin (5.45%), emodin-glucoside (0.097%), emodin (0.049%), chrysophanol (0.464%)	[9]
50	70% ethanol extract	–	–	UPLC/Q-TOF–MS/MS; chromatographic fingerprints	Aloe-emodin, rhein, emodin, chrysophanol, physcion, catechin, gallic acid, methyl gallate, ethyl gallate, torachrysone, hydroxy physcion, 3,5,4’-trihydroxy-transstilbene, sennoside A, solindleyin, ect	[136]
51	Methanol extract	*R. officinale*; *R. palmatum; R. emodi; R. tanguticum*; *R. franzenbachii; R. hotaoense;*	Root	Liquid chromatography coupled with electrospray ionization tandem mass spectrometry	Aloe-emodin, rhein, emodin, chrysophanol, physcion, emodin 8-O-glucoside, chrysophanol 8-O-glucoside, sennoside A, rhaponticin, *epi*-catechin, ect	[16]
52	Methanol extract	*R. palmatum* L.	–	Ultra-performance liquid; PLC/Q-TOF–MS	Emodin-8-O-glucoside, emodin-O-glucoside, catechin-glucopyranoside, gallic acid-3-O-glucoside, torachrysone, chrysophanol dimethylether	[137]
53	70% methanol extract	*R. palmatum* L.	–	LC–QTOF MS	Rhapontigenin, rhein, resveratrol, cinnamoyl, chrysophanol, physcion, emodin, aloe-emodin, anthranone, dianthrone, etc	[138]
54	Water–acetone extract	*R. rhabarbarum* L.	Stalk	LDI-MSI with gold nanoparticle -enhanced target (AuNPET)	Emodin, aloe-emodin, chrysophanol, citreorosein, citreorosein, sennoside E and F, resveratrol, trans-stilbene, rhaponticin, piceatannol 4’-galloylglucoside, quercetin, oxalic acid, succinic acid, carvacrol, dehydroascorbic acid, pcoumaric acid, lysine, chromene, paeonol, vitamin B5 and D1, amino acids threonine, phenylalanine, digalloyl-substituted, procyanidin B2 and B5, gallic acid, magnesium oxalate, ect	[139]
55	Ethanol extract	*R. palmatum* L.	–	HPLC coupled with photodiode array detector	Gallic acid, catechins, epicatechin gallate, chrysophanol (> 0.07%), epigallocatechin gallate, epicatechin, sennoside A, sennoside B, aloe-emodin, rhein, emodin, physcion (< 0.04%)	[140]
56	70% methanol extract	*R. tanguticum* Maxim.ex Balf.	–	HPLC-LTQ-Orbitrap-MS	Emodin, rhein, physcion, aloe-emodin, chrysophanol, (+)-catechin, sennoside A, sennoside B, sennoside C, polydatin, gallic acid, emodin- 8-O-β-d-glucopyranoside, aloe-emodin-8-O-glucoside, epicatechin-3-O-gallate, etc	[141]
57	Methanol extract	*R. tanguticum* Maxim.ex Balf.	–	UPLC/Q-TOF–MS/MS	p-coumaric acid glucoside, (−) epicatechin-3-O-gallate, isolindleyin, phlorizin, emodin, aloe-emodin, emodin-1-O-β-d-glucopyranoside, 4-(4′-hydroxyphenyl)-2-butanone 4′-O-β-d-(6-O-trans-p-coumaroyl)-glucopyranoside, resveratrol-4′-O-β-d-(2′′-O-galloyl)-glucopyra-noside, etc	[142]
58	Methanol extract	–	–	UHPLC	Sennoside A, physcion, rhein, chrysophanol, aloe-emodin, emodin, aloe-emodin-8-O-β-d-glucopyranoside, rhein-8-O-β-d-glucopyranoside, emodin-8-O-β-d-glucopyranoside, chrysophanol-8-O-β-d-glucopyranoside, physcion-8-O-β-d-glucopyranoside	[143]
59	80% methanol extract	*R. palmatum* L.; *R. tanguticum* Maxim.ex Balf.; *R. Officinale* Baill.	–	HPLC coupled with PDA	Physcion, rhein, aloe-emodin, emodin, chrysophanol, gallic Acid, sennoside A, sennoside B, catechins, epicatechin, epigallocatechin gallate, epicatechin gallate	[144]

Metabolomic characterization or metabolite profiling has been used to analyze TCMs and screen for bioactive markers. Based on absorption, distribution, metabolism and excretion characteristics, these methods aim to detect representative profiles of metabolites for different species [11]. Tseng et al. developed ultrahigh-pressure LC (UHPLC) methods coupled with UV detectors to characterize the metabolomic profiles of different rhubarb species [13]. It is noteworthy that this method can be used for hybrid rhizome detection due to its high sensitivity and selectivity. Small chemical constituents in herbs originating from hybridization can be clearly observed through similarity measurements of metabolic profiles. Although fingerprint analysis by HPLC and metabolite profiling by chromatographic-mass spectrometric methods have high sensitivity and selectivity, long runtime and expensive instruments limit their broad use. The chloroplast gene sequence can be used as a quality evaluation index of rhubarb, which may indicate the production area of the plants. However, the identification of each species has not been clarified [17].

In conclusion, identification by HPLC coupled with detectors based on the main chemical constituents of rhubarb has become the mainstream method. Chemical fingerprint analysis methods reflect the majority of components in rhubarb, which is one of the most accurate and sensitive identification methods at present and can even reflect the origin of rhubarb. The metabolomic characterization method can identify the hybrid rhizome, and compared with fingerprint analysis by HPLC, the analytical time is shorter. Genomics has been introduced to study TCMs, which may suggest the production areas of plants, but some limitations should be noted: plants growing in neighboring areas have the same or similar mature enzyme gene of the chloroplast genome (matK gene) sequences, so the identification of different species in adjacent areas can be confusing.

## Pharmacology

**Fig. 2:**
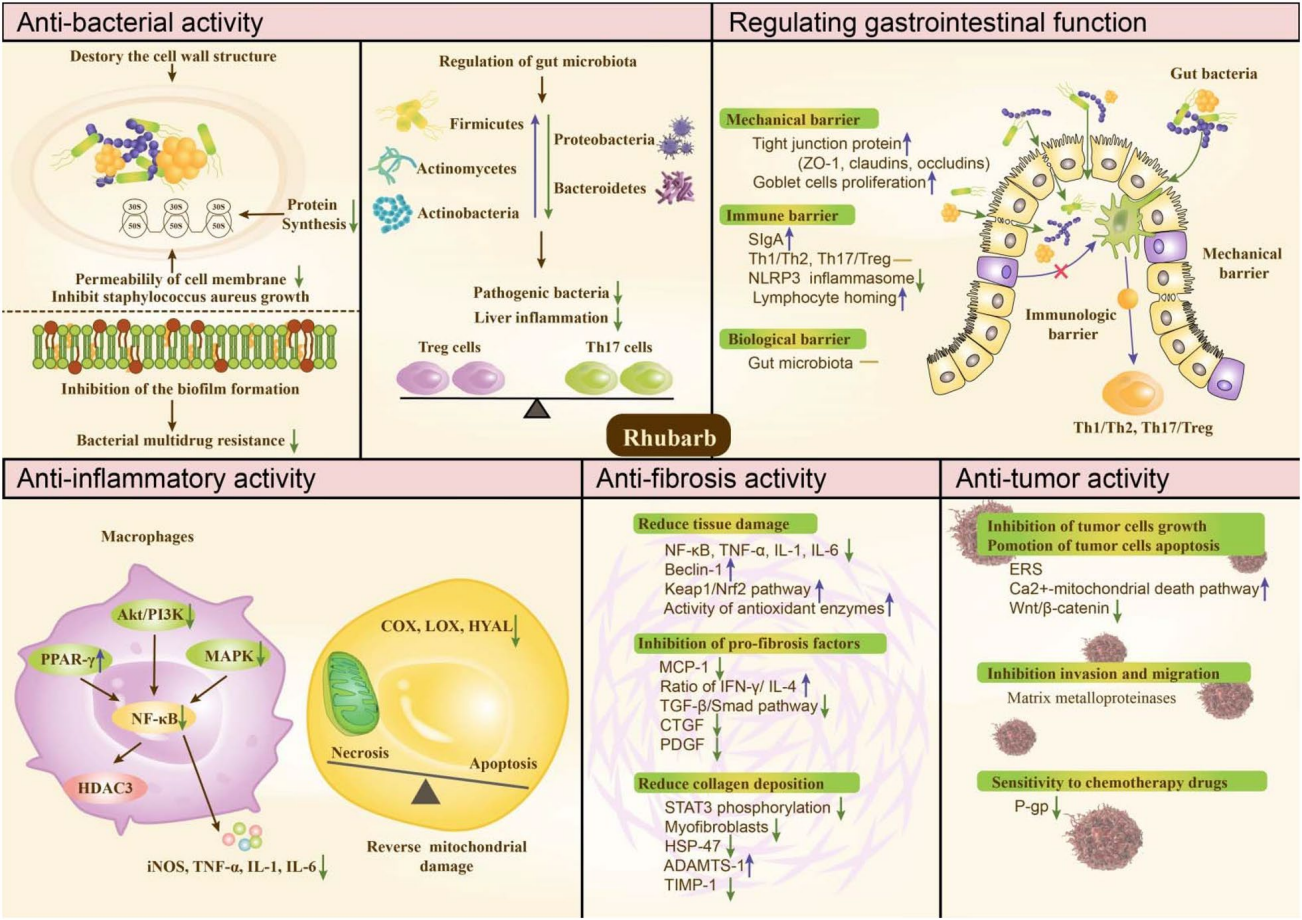
Overview of the pharmacological activity of rhubarb

With the development of a research focus on rhubarb, an increasing number of pharmacological effects have attracted the attention of researchers. The main pharmacological activities of rhubarb include antitumor [18], regulation of gastrointestinal flora [19], protection of the intestinal mucosal barrier [5, 6], anti-inflammatory [20], and inhibition of fibrosis [21]. In addition to emodin and sennoside, which are the main chemical components inhibiting fibrosis and purgation, most of the pharmacological effects are the result of the joint action of several anthraquinones in rhubarb. Figure 2 shows an overview of the pharmacological activities and functional mechanisms of rhubarb.

### Regulation of bacterial action

Rhubarb has efficient antibacterial activities against a variety of bacteria, including *Staphylococcus aureus*, bifidobacteria, Lactobacillus, *Helicobacter pylori*, *Escherichia coli*, methicillin-resistant *Staphylococcus aureus* and multidrug-resistant *Helicobacter pylori* [22, 23]. It is noteworthy that rhubarb methanol extract has stronger inhibition of *E. coli*, *Listeria monocytogenes*, *Klebsiella pneumoniae* and *Bacillus subtilis* than standard antibiotics [24]. Mechanistically, rhubarb can inhibit the growth of *Staphylococcus aureus* by destroying its bacterial cell wall structure and changing the permeability of the cell membrane [25]. The formation of bacterial biofilms is an important cause of bacterial multidrug resistance. *Streptococcus suis* is one of the most important swine pathogens and can cause persistent infection in humans. Ding et al. showed that rhubarb can inhibit the formation of biofilms by downregulating transduction systems, influencing the levels of DNA binding protein and transcriptional regulation factors [26]. Similarly, emodin can inhibit the biofilm formation of *Pseudomonas aeruginosa* and *Stenotrophomonas maltophilia* [27]. Aloe-emodin can destroy bacterial membranes by the interaction between phosphatidylethanolamine and phosphatidylglycerol [28].

In addition, the effect of rhubarb on the human gut microbiota is complex and diverse. The homeostasis of gut microbiota is conducive to inhibiting the growth of pathogenic bacteria in the intestinal tract [29] and maintaining host energy, metabolic homeostasis, and the immune system [30, 31]. Zhang et al. pointed out that the number of intestinal *Escherichia coli*, bifidobacteria and total anaerobes increased after rhubarb was given to critically ill patients [19]. Luo et al. found that TCM prescriptions containing rhubarb increased the number of intestinal Firmicutes and actinomycetes [32]. Moreover, studies have shown that rhubarb could restore the Th17/Treg balance by restoring diversity, significantly increasing the abundance of Firmicutes and Actinobacteria and decreasing Proteobacteria and Bacteroidetes [32, 33]. Rhubarb can also improve ulcerative colitis in mice by regulating gut microbiota to restore Th17/Treg balance [32]. In addition, rhubarb can prevent liver inflammation caused by acute alcohol intake [34] and change the content of aromatic amino acids and phenol in feces by regulating gut microbiota [35].

Overall, rhubarb has strong antibacterial activity and a wide antibacterial spectrum, and it can also reverse bacterial resistance to antibiotics by inhibiting the formation of bacterial biofilms. Oral rhubarb has intervention effects on the ecological balance of intestinal flora, thus playing a positive role in inflammation, immunity and other aspects of the body.

### Regulating gastrointestinal function

The main medicinal effect of rhubarb on the gastrointestinal tract is to promote the digestion of residual food. Anthraquinone compounds in rhubarb, including sennosides, rheinosides and anthraquinone aglyconesa, have efficient laxative effects by promoting intestinal contraction and movement [2]. Furthermore, anthraquinone compounds can stimulate the submucosal nerve plexus, reduce the conductance of K^+^ channels on the cell membrane and enhance the electrical excitability of intestinal smooth muscle cells. In addition, tannins extracted from rhubarb have been proven to play an antidiarrheal role, and the underlying mechanism may be its promotion of protein coagulation [36].

The intestinal mucosal barrier is composed of the epithelial barrier, immune barrier, intestinal flora barrier and chemical barrier [37], which have the function of separating the contents in the intestinal cavity and preventing the invasion of pathogenic antigens [38]. Many clinical and animal experiments have proven that rhubarb could protect the intestinal mucosal barrier, and its mechanism is complex: (1) Rhubarb can maintain the balance of gut microbiota, make the disturbed flora return to normal and prevent the gut microbiota from shifting [5, 6]. (2) Rhubarb ameliorates mucosal damage through modulating intestinal permeability by increasing the expression of junction proteins [39]. (3) Rhubarb can regulate the immune function of the intestine. Briefly, rhubarb alleviates excessive innate immune- mediated inflammatory responses and intestinal damage by inhibiting the expression of the nucleotide-binding oligomerization domain-like receptor protein 3 (NLRP3) inflammasome [40]. Moreover, rhubarb anthraquinones can increase the expression of secreted immunoglobulin A (SIgA) and restore the balance of Th1/Th2 and Th17/Treg [40, 41]. In addition, rhubarb can regulate intestinal lymphocyte homing, improve the immune function of intestinal mucosa and relieve ulcerative colitis [42]. (4) Rhubarb promotes the proliferation of goblet cells in the intestinal mucosa, which can secrete a large amount of mucus to reinforce the intestinal mucosal barrier by forming a mucosal layer [34].

In short, there are a variety of pharmacological effects of rhubarb in the gastrointestinal tract. The combination of anthraquinones in rhubarb play a role in inducing diarrhea, while tannic acid can inhibit the laxative effect of anthraquinones and thereby induce antidiarrheal effects. Moreover, rhubarb can maintain or restore the barrier function of the intestinal mucosa and prevent gut microbiota from shifting. Rhubarb also has the function of regulating gastrointestinal motility disorders [19, 43], increasing intestinal blood perfusion [5], clearing gastrointestinal oxygen free radicals, and eliminating inflammatory factors [34].

### Anti-inflammatory activity

In recent years, the underlying mechanisms of the anti-inflammatory effects of rhubarb have attracted more attention. Kolodziejczyk-Czepas et al. reviewed the anti-inflammatory effects of rhaponticin and the aglycone rhapontigenin and concluded that their anti-inflammatory effects were induced by inhibiting cyclooxygenase (COX), lipoxygenase (LOX) and hyaluronoglucosaminidase (HYAL) activation and modulating a variety of pro-inflammatory responses [20]. In addition to rhaponticin, various other components of rhubarb have anti-inflammatory effects, such as emodin, rhein, chrysophanol, and aloe emodin.

Wen et al. constructed an inflammation model by stimulating RAW264.7 cells with lipopolysaccharide (LPS) and treated the cells with rhein. These results showed that rhein exerts its anti-inflammatory function by regulating the peroxisome proliferator-activated receptor-γ/nuclear factor kappa B/histone deacetylases 3 (PPAR-γ/NF-κB/HDAC3) axis [44]. Sha et al. demonstrated that rhein ameliorates radiation-induced acute enteritis in vivo through the same pathway [45]. In addition, emodin and chrysophanol could inhibit LPS-induced inflammation in RAW264.7 cells through the PPAR-γ-dependent pathway [46, 47]. Hu et al. treated LPS-stimulated RAW264.7 macrophages with aloe-emodin (5–20 μM) to investigate the anti-inflammatory effects of aloe-emodin [48]. The results showed that aloe-emodin at 10 μM or 20 μM exerts anti-inflammatory effects by reducing the activation of NF-κB via the inhibition of inhibitor of NF-κB-α (IκBα) degradation and mitogen-activated protein kinase (MAPK) phosphorylation. Feng et al. treated acute pancreatitis (AP) rats with rhubarb decoction orally (150 mg/kg) and found that rhubarb probably attenuated AP by inhibiting activation of MAPKs [49]. Our previous studies also proved that emodin alleviated AP via miRNA-30a-5p/HtrA serine peptidase 1/transforming growth factor-β (HTRA1/TGF-β) and purinergic receptor P2X, ligand-gated ion channel, 7/NLRP3 (P2X7/NLRP3) inflammatory signaling [50, 51].

In the treatment of inflammatory diseases, rhubarb can promote recovery of the structure and physiological functions of various organs and improve the cure rate and prognosis of patients. For instance, emodin can reduce the degree of acinar necrosis and induce apoptosis in rats with acute necrotizing pancreatitis [52]. In addition, chrysophanol can reverse mitochondrial damage, promote acinar cell proliferation, and reduce the degree of damage to pancreatic tissues [53].

### Anti-fibrotic activity

Fibrosis is the common outcome of chronic liver injury, chronic kidney disease, pulmonary interstitial disease and other chronic diseases. Degeneration and necrosis of parenchymal cells, as the primary causes of fibrosis, can activate macrophages and release active factors. In conditions with active factors, static extracellular matrix (ECM)-producing cells activate into myofibroblasts, which can increase the synthesis of ECM and eventually cause fibrosis [54]. Rhubarb alleviates fibrosis by inhibiting or reversing the necrosis of parenchymal cells, reducing the activation and migration of monocytes to damaged tissues, inhibiting the activation of fibroblasts, relieving collagen deposition, and promoting the degradation of collagen.

The role of the inflammatory response in the development and progression of fibrosis has been recognized. On the one hand, inflammation damages parenchymal cells; on the other hand, it activates corresponding macrophages and releases active factors to promote the occurrence and progression of fibrosis [55]. Tian et al. treated bleomycin-induced pulmonary fibrosis in rats with emodin (20 mg/kg) and found that emodin protected rats from pulmonary fibrosis by inhibiting the activation of NF-κB and reducing the expression of pro-inflammatory factors, such as tumor necrosis factor-α (TNF-α), interleukin-6 (IL-6) and interleukin-1β (IL-1β) [21]. Emodin also inhibits the expression of monocyte chemoattractant protein (MCP-1) to reduce the infiltration of monocyte-derived macrophages into the liver. Cytokines secreted by macrophages and lymphocytes play important roles in the pathogenesis of fibrosis [56]. Reducing the ratio of IFN-γ/IL-4 is beneficial to the occurrence and development of pulmonary fibrosis, while increasing the ratio of IFN-γ/IL-4 can alleviate pulmonary fibrosis [57, 58]. Furthermore, emodin can reduce the expression of collagen I (Col I), collagen III (Col III) and Beclin 1 and thereby improve the degree of renal fibrosis [59].

TGF-β is the most powerful fibrogenic cytokine that can secrete ECM by directly activating a variety of ECM-producing cells and reducing ECM degradation by stimulating the generation of protease inhibitors [60]. The Smad protein family includes the most important downstream molecules in the intracellular signaling process of the TGF-β superfamily. A recent study has shown that emodin alleviates pulmonary fibrosis by increasing the expression of Smad7 and thereby silencing the TGF-β/Smad pathway [61]. Guan et al. found that emodin could inhibit the activation of myofibroblasts by downregulating the phosphorylation of signal transduction and transcriptional activator-3 (STAT3) and further decrease the synthesis of collagen by reducing the expression of heat shock protein-47 (HSP-47) [5, 62]. Similarly, emodin also alleviates renal fibrosis by inhibiting the expression of connective tissue growth factor (CTGF) and platelet-derived growth factor (PDGF) and the deposition of Col I and Col III [63]. In addition, emodin can also increase the degradation of Col I and Col III and ECM in lung tissues by upregulating the expression of a disintegrin-like and metalloproteinase with thrombospondin type 1 motif (ADAMTS-1) and downregulating the expression of tissue inhibitor of metalloproteinase-1 (TIMP-1), respectively [64, 65]. In conclusion, emodin exerts antifibrotic effects by regulating the inflammatory response, oxidative stress, autophagy, and TGF-β–mediated ECM processes.

### Antitumor activity

Rhubarb has a strong inhibitory effect on a variety of tumors in the digestive, respiratory and reproductive systems, such as human stomach cancer [66], pancreatic cancer [67], nasopharyngeal carcinoma, lung adenocarcinoma [68] and ovarian carcinoma [69]. Rhubarb plays vital roles in multiple biological processes of tumor development through several pathways and targets. It inhibits the growth of tumor cells [18], suppresses tumor invasion and migration [69, 70], and even hinders the formation of tumor neovascularization. These antitumor responses may be attributed to a variety of antitumor chemicals extracted from rhubarb, such as emodin, rhein, aloe emodin, and distyrene [2]. A previous study found that rhein could induce human aortic smooth muscle cell apoptosis via a mitochondria-dependent pathway but mediate human nasopharyngeal carcinoma cell apoptosis through an endoplasmic reticulum stress- and Ca^2+−^dependent mitochondrial death pathway. In addition, rhein inhibited the migration and invasion of human tongue cancer SCC-4 cells and human ovarian carcinoma SKOV3-PM4 cells by modulating matrix metalloproteinases [69, 70]. These findings suggest that the chemical monomers of rhubarb affect many biological processes related to cancer through multiple pathways. The Wnt/β-catenin signaling pathway plays an important role in cell proliferation and invasion in lung cancer, stomach cancer, ovarian cancer and nephroblastoma. Previous studies have shown that rhubarb also inhibits tumor metastasis by promoting the degradation of β-catenin protein and negatively regulating the Wnt signaling pathway [3]. It is worth mentioning that rhubarb can inhibit the invasion and metastasis of lung cancer, gastric cancer and ovarian cancer. Whether this is related to rhubarb’s influence on the Wnt/β-catenin signaling pathway remains to be proven.

Compared with traditional cytotoxic drugs, it is very difficult for tumors to develop resistance to rhubarb due to its multiple targets. Interestingly, emodin at a concentration of 10 µM can increase the sensitivity of tumor chemotherapy and radiotherapy by decreasing the function of P-glycoprotein (P-gp) and activating the mitochondrial apoptosis pathway in vitro [71, 72]. Although numerous studies have demonstrated that rhubarb can inhibit multiple biological processes and signaling pathways in tumors, few clinical applications have been reported. This may be due to the low cytotoxicity selectivity of rhubarb at high doses. Studies have shown that the monomer components of rhubarb had significant cytotoxicity to human normal liver LO2 cells, HL-7702 cells [73] and human renal tubular epithelial HK-2 cells [74, 75] in a dose- and time-dependent manner. Therefore, more attention should be paid to developing targeted agents from rhubarb to reduce the distribution of drugs in noncancerous tissues.

## Clinical applications

Inflammation, infection and oxidative stress are the most common causes of cell damage. Due to its extensive pharmacological effects, rhubarb can interfere with the development of various diseases. The majority of studies have focused on the efficacy of rhubarb on severe acute pancreatitis (SAP) [5, 6], sepsis [7], chronic renal failure (CRF) [8], etc. The results are summarized in Table 3. Most clinical experiments with rhubarb and its active constituents adopt randomized, double-blind and placebo-controlled trials as the study design, and the results have shown that rhubarb could significantly improve feeding tolerance in critically ill patients, intestinal permeability in sepsis patients, oxygenation function in acute respiratory distress syndrome (ARDS) patients, and so on. Specifically, rhubarb has a significant effect on SAP patients. It can effectively relieve abdominal pain, abdominal distension and other symptoms, shorten the ICU and hospital stay of SAP patients by promoting the absorption of pancreatic exudate, improving gastrointestinal function, reducing systemic inflammation and mitigating SAP-related damage to the liver and kidney.

**Table 3:** Clinical trials of Rhubarb and the active constituents

No.	Drugs	Species	Subjects	Study design	Outcome	Mechanism	Quality of evidence	Refs.
1	Crude rhubarb	–	368 critically ill patients with Grade I–III AGI	Retrospective study based on propensity score matching	Rhubarb significantly improve feeding tolerance and relieve gastrointestinal dysfunction in critically ill patients without serious adverse reactions	CRP ↓	III	[19]
2	Rhubarb powder	–	112 critically ill patients with IAH and AGI stage I-III	Randomized trial	Rhubarb enema had a protective effect against IAH and may be more effective compared with glycerin enema	PCT ↓, CRP ↓, TNF-α ↓, IL-6 ↓, ventilation function ↑	IIa	[145]
3	Rhubarb decoction (50 g rhubarb slice plus 100 ml water)	–	94 Chinese patients who required jejunal feeding tube insertion	Randomized trial	Significantly shorter insertion time in the rhubarb group	–	IIa	[146]
4	Rhubarb syrup	*R. ribes* L.	150 Iranian children aged between 12-72 months with suspected Shigella dysentery	Randomized, double-blind, placebo-controlled trial	Significant improvement of symptom and shorter duration of need for antipyretics	–	Ib	[147]
5	Rhubarb	–	912 participants with SAP	Meta-analysis for a total of 16 randomized controlled trials	Rhubarb plus trypsin inhibitor group showed lower mortality, length of hospitalization, abdominal pain relief time, and serum amylase level	–	Ia	[148]
6	Rhubarb combined with early EN	–	126 patients with SAP	A randomized controlled trial	Significant improvement of symptom, renal function and shorter periods of hospital stays in the EEN/rhubarb group	–	Ib	[149]
7	Rhubarb combined with mirabilite	–	96 patients with SAP	A randomized controlled trial	The times taken for abdominal distension, abdominal pain, blood and urinary amylase values to normalize were significantly shorter	WBC count ↓, CRP ↓, IL-6 ↓	IIa	[80]
8	Crude rhubarb combined with somatost-atin	–	1161 patients with acute pancreatitis	Meta-analysis of 19 randomized controlled trials	Adjuvant treatment with crude rhubarb appears to have additional benefits. Reduced the total complications and duration of hospital stay	Improve the gastrointestinal function	Ia	[150]
9	Raw Rhubarb Solution	–	500 Chinese patients with high-risk factors of pancreatitis after post-ERCP	Predictive random compared research in one center	Raw rhubarb significantly reduced the incidence of post-ERCP pancreatitis in high-risk patients	–	IIa	[151]
10	Crude rhubarb	*R. palmatum* L.	869 patients with systemic inflammation reaction syndrome/sepsis	Meta-analysis of 15 randomized controlled trials	Crude rhubarb has additional benefits in the treatment of sepsis; improve the gastrointestinal function	IL-6 ↓, TNF-α ↓, prothrombin time ↓ platelet number ↑	Ia	[152]
11	Crude rhubarb	–	40 septic patients with APACHE II score of above 12	A randomized controlled trial	Crude rhubarb can improve intestinal permeability in patients with sepsis	Procalcitonin level ↓	Ib	[84]
12	Rhubarb capsule	–	Patients with chronic kidney disease (stages 3 & 4) of age 20–60 years	A prospective comparative study	Significantly improve the clinical features and biochemical parameters	–	Ib	[153]
13.	Capsules of Rhubarb stem extract	–	80 Iranian patients with type II diabetes mellitus aged 30–60 years old with fasting blood glucose greater than 140 mg/dl	Randomized, double-blind, placebo-controlled trial	Significant reduction of HbA1C and fasting blood glucose with rhubarb intervention	–	Ib	[154]
14	Rhubarb powder (Yalan Pharmaceutical Co., Ltd., Lanzhou, China)	–	85 patients with heat stroke	Randomized controlled trials	Significant improvemen of liver and kidney function, shorter ICU and hospital stays, and a lower APACHE II score in the rhubarb group	WBC ↓, CRP ↓, PCT ↓, IL-6 ↓	IIa	[155]
15	Rhubarb extract	–	125 patients with minor aphthae	Randomized, placebo-controlled trial	Symptom resolution time: rhubarb (1.84 days), placebo (4.64 days)	–	IIa	[156]
16	Rhubarb leachate (extracted with 30 mL of boiling water)	–	80 Chinese patients aged 22–79 years with ARDS	Randomized, controlled trial	Rhubarb can decrease EVLWI and PVPI, and improve oxygenation in patients	–	IIa	[157]
17	R. emodi powder capsule (420 mg of powdered drug per capsule)	*R. Emodi* L.	45 unmarried participants aged 15–25 years having regular menstrual cycles with dysmenorrhoea	Randomized, single-blind, standard controlled trial	Improve in dysmenorrhoea quality of life	–	IIa	[158]
18	Crude rhubarb	*R. palmatum* L	886 Chinese patients with AOPP	Meta-analysis of a total of 12 randomized controlled trials	Reduce the incidence of intermediate syndrome and MODS, total dose of pralidoxime or atropine	Restore cholinesterase function and purgative	Ia	[159]
19	Rhubarb extract (extracted with water at 100 °C for 4.5 h)	–	A total of 80 patients with lung cancer	A randomized, double-blind, placebo-controlled trial	Rhubarb extract significantly decrease attenuated radiation induced lung toxicity (RILT) and improve pulmonary function	TGF-β1 ↓, IL-6 ↓	Ib	[160]
20	*R. Officinalis* Baill (extracted with water)	*R. Officinalis* Bail.	103 patients with athero- sclerosis aged from 45 to 65 years	A randomized, double-blind, placebo-controlled clinical trial	Significantly improves endothelial function in patients with atherosclerosis	TC ↓, LDL-C ↓, FMD ↑	Ib	[161]
21	Prepared rhubarb	–	92 patients with PIH	A randomized controlled trial	Improve IR of PIH	Regulation of GLUT1	Ib	[162]

### Constipation

Rhubarb contains a large number of anthraquinone chemical constituents, which have a strong purgatory function. Rhubarb powder and peppermint oil are commonly used to treat constipation caused by thoracolumbar fracture [4], type 2 diabetes or acute stroke [76]. A meta-analysis of 850 constipation patients based on 10 randomized controlled trials (RCTs) showed that the regimen was effective in treating constipation, and no serious adverse events were reported in any trial [77].

### Severe acute pancreatitis

SAP is a common clinical acute abdominal disease with a mortality rate as high as 20%-30% [5]. A large number of clinical studies have shown that rhubarb enema can reduce serum inflammatory cytokines, high sensory C-reactive protein (CRP) and endotoxin levels, and relieve the systemic inflammatory stress response and restore intestinal mucosal barrier function in SAP patients [78, 79]. Moreover, applying hot compresses with rhubarb decreases the symptoms of pancreatic leakage in SAP patients [80]. Meanwhile, for SAP patients with gastrointestinal retention and paralysis, nasal injections of rhubarb can also achieve therapeutic effects [81]. In addition, capillary ischemia, blood stasis, microthrombus formation and other microcirculation disorders are common in the early stage of SAP, and rhubarb can reverse the decline in pancreatic blood flow and reduce pancreatic bleeding, which may be related to its anti-inflammatory, blood circulation promotion and blood stasis removal effects [49].

Clinically, the combination of rhubarb and the basic treatment for SAP is commonly used to increase the therapeutic effect. Two meta-analyses involving nearly 2000 SAP patients were conducted to evaluate the efficacy and safety of trypsin inhibitors or somatostatin combined with rhubarb in the treatment of SAP, and the results showed that this treatment can significantly reduce hospital stay, mortality and serum amylase levels in SAP patients [5, 6]. Moreover, rhubarb was used in combination with early enteral nutrition [6], high-volume hemofiltration [82], and magnesium sulfate [7]. These combinations significantly decreased the severity and levels of liver and kidney damage in SAP patients by improving gastrointestinal function and decreasing the systemic inflammatory response. Therefore, rhubarb alone or rhubarb combined with the basic treatment of SAP may be safe and effective treatments for patients.

### Sepsis

Sepsis is a systemic inflammatory response syndrome (SIRS) caused by infection, and the gastrointestinal tract is an important target organ for promoting SIRS after infection [83]. Sepsis patients often suffer from immune dysfunction and abnormal coagulation function. A meta-analysis sorting out the treatment information of 869 patients in 15 RCTs showed that rhubarb was effective in the adjuvant treatment of sepsis [7]. Prothrombin time and proinflammatory factor (such as IL-6 and TNF-α) levels significantly decreased and platelet count markedly increased after rhubarb administration. However, crude rhubarb treatment did not significantly reduce 28-day mortality compared to conventional treatment [7]. In addition, patients with SIRS were treated with rhubarb powder by oral administration or nasal feeding for three days, and the results showed that the levels of serum TNF-α, CRP and complement 3 (C3) and 4 (C4) dramatically decreased. Another randomized double-blind experiment randomly selected 40 eligible sepsis patients and found that rhubarb can reduce intestinal mucosal permeability in patients with sepsis, thereby reducing bacterial toxin translocation and alleviating symptoms in patients with sepsis [84, 85]. These results suggest that rhubarb may be used in the clinical treatment of sepsis via its anti-inflammation, anticoagulation, gastrointestinal protection, and bacterial and toxin translocation inhibition effects. In addition, rhubarb can also increase T cell subsets, which suggests that the regulation of immune function may also contribute to the mechanism of rhubarb in treating sepsis [83].

### Chronic renal failure

The common pathology of CRF is renal fibrosis, which involves the glomerulus and renal interstitium [86]. Rhubarb has unique advantages in improving the early symptoms of CRF and delaying the progression of renal failure [8]. The potential mechanisms include inhibition of renal fibrosis, promotion of toxin excretion, recovery of metabolic disorders, protection of renal cells from excessive inflammation and oxidative stress damage [87]. Rhubarb is mainly administered by retention enema in the treatment of CRF, and the dose should depend on the number of defecation times of the patient per day [88]. Shenkang injection is a kind of TCM that is extracted and refined rhubarb, astragalus, *Salvia miltiorrhiza* and safflower. A phase IV clinical study included 2200 subjects, and the total effective rate was 73.05% after Shenkang injection treatment for renal failure [89].

### Others

Oral rhubarb rhizome extract significantly reduced glycosylated hemoglobin, fasting glucose, and body weight in patients with type 2 diabetes [90]. Rhubarb liquid nasal infusion combined with montmorillonite powder and blood purification can rapidly remove the toxins in patients with organophosphorus pesticide poisoning, reduce adverse reactions and shorten the length of stay in the hospital [91, 92]. Meta-analysis showed that the adjuvant treatment of organic phosphorus pesticide poisoning with crude rhubarb could significantly reduce the incidence of intermediate syndrome and multiple organ dysfunction syndrome [64]. Clinical studies have shown that the levels of venous white blood cells (WBCs), CRP, procalcitonin (PCT) and IL-6 in patients with heatstroke were reduced significantly after treatment with rhubarb supplementation (0.3 g/kg body weight) for 3–5 days. Rhubarb can also treat respiratory distress syndrome [93], cholestatic hepatitis [94], hepatic encephalopathy [95], among others.

## Safety issue

Preclinical studies have shown that rhubarb has toxic effects on the liver and kidneys and is associated with cancer risk. Emodin, the main causative agent of rhubarb hepatotoxicity [96], can cause apoptosis in normal human L02 cells and increase the expression of liver injury markers [97]. In addition, emodin can affect the oxidative phosphorylation pathway by inhibiting the activity of all mitochondrial complexes, which causes mitochondrial damage, decreases in mitochondrial membrane potential (MMP), increases in reactive oxygen species (ROS), adenosine triphosphate (ATP) synthesis disorder, and finally liver cell apoptosis [98]. In addition, oral rhubarb or rhubarb products pose a risk of nephrotoxicity due to the abundance of oxalates and anthraquinones, which can lead to deterioration of kidney function as a result of oxalate excretion disorder and crystal deposition in the kidney [99]. However, renal dysfunction due to the above causes has been reported only in children or patients with mild renal disease who have been taking large amounts of rhubarb for a long time [100]. Animal studies have shown that high-dose treatment with rhubarb anthraquinones causes changes in the expression of MAPK kinase 6 and cytochrome P4501A1 (CYP1A1), leading to the swelling and denaturation of renal tubular epithelial cells [101]. It is important to note that the kidney changes were only triggered when the dose was 600 times higher than the clinical dose, suggesting that kidney damage from rhubarb anthraquinone is negligible.

Whether rhubarb has a cancer risk has been controversial for nearly 30 years. A large number of preclinical studies have shown that rhubarb anthraquinones have mutagenic and genotoxic effects [102], and long-term administration of anthraquinone laxatives may damage epithelial cells and induce gastric cancer and colorectal cancer [103]. However, few studies evaluating the possible carcinogenic effects of anthranoid laxatives have been performed in humans [104]. A retrospective analysis of drug use and gastric cancer in 14,616 patients also showed that the use of rhubarb may have little relationship with the development of gastric cancer in practice [105]. In addition, a meta-analysis proposed a relationship between rhubarb anthraquinones and colorectal cancer, but the results have not been examined.

In conclusion, taking rhubarb may damage the health of the liver, and it is recommended to reduce the use of rhubarb in patients with inflammation in the liver due to illness or medication. Long-term use of rhubarb has an effect on liver drug enzyme UDP-glucuronosyltransferase 2B7 (UGT2B7) and transporter multidrug resistance protein 2 (MRP2), so it is necessary to pay attention to the effect on metabolism and excretion of the same drug to prevent adverse effects caused by drug interactions. Patients with kidney disease should carefully avoid long-term use of rhubarb. Whether rhubarb causes cancer remains controversial. Although preclinical studies have shown a carcinogenic risk for rhubarb anthraquinone, the results of animal studies should be carefully generalized to applicability in humans due to the high doses used in animal studies and the relatively long duration of use compared to the animal’s lifespan [106].

## Conclusion and perspective

Rhubarb is widely distributed across Europe, North America and part of Asia, and the species from different origins are obviously different. Much effort has been focused on the identification of these various species in recent years. Among them, chemical fingerprint and metabonomic identification methods can reflect the origin and hybrids of rhubarb, respectively. Rhubarb has been used to interfere with the development of various diseases, including SAP, sepsis, and CRF, due to its antibacterial, anti-inflammatory, and antifibrotic activities and the regulation of gastrointestinal function. However, several critical issues need to be considered in future studies. Rhubarb has significant hepatotoxicity, which may be related to the accumulation of emodin in the liver or its influence on the oxidative phosphorylation pathway, and gender may also be a factor affecting its hepatotoxicity. The mechanisms underlying rhubarb hepatotoxicity remains unclear, but understanding the hepatotoxicity is of great value for the clinical promotion of rhubarb. It remains controversial whether long-term use of rhubarb can cause cancer. In addition, the existing literature on the clinical application of rhubarb is mostly based on the experience of doctors and lacks uniform standards. The species and origin of rhubarb used in most of these studies have not been reported, and the cases included in the analyses lack information regarding age, race and other aspects of the participants. More scientific, rigorous and extensive clinical trials are needed to gain insight.

## Data Availability

All data included in this article are available from the corresponding author upon request.

## Abbreviations

TCM: Traditional Chinese medicine
TLC: Thin-layer chromatography
HPLC: High-performance liquid chromatography
CZE: Capillary zone electrophoresis
MEC: Micellar electrokinetic chromatography
UV: Ultraviolet
DAD: Diode array detector
CE: Capillary electrophoresis
MS: Mass spectrum
HPLC–DAD: High-performance liquid chromatography-photodiode array detection
UHPLC: Ultra-high-pressure LC
NLRP3: Nucleotide-binding oligomerization domain-like receptor protein 3
SIgA: Secreted immunoglobulin A
COX: Cyclooxygenase
LOX: Lipoxygenase
HYAL: Hyaluronoglucosaminidase
LPS: Lipopolysaccharide
PPAR-γ: Peroxisome proliferator-activated receptor-γ
NF-κB: Nuclear factor kappa B
HDAC3: Histone deacetylases 3
MAPK: Mitogen-activated protein kinase
AP: Acute pancreatitis
HTRA1: HTRA serine peptidase 1
TGF-β: Transforming growth factor-β
P2X7: Purinergic receptor P2X, ligand-gated ion channel, 7
ECM: Extracellular matrix
TNF-α: Tumor necrosis factor-α
IL-6: Interleukin-6
IL-1β: Interleukin-1β
MCP-1: Monocyte chemoattractant protein
IFN-γ: Interferon-γ
IL-4: Interleukin-4
Col I: Collagen I
Col III: Collagen III
STAT3: Signal transduction and transcriptional activator 3
HSP-47: Heat shock protein-47
CTGF: Connective tissue growth factor
PDGF: Platelet-derived growth factor
ADAMTS-1: A disintegrin-like and metalloproteinase with thrombospondin type 1 motif
TIMP-1: Tissue inhibitor of metalloproteinase-1
P-gp: P-glycoprotein
SAP: Severe acute pancreatitis
CRF: Chronic renal failure
ARDS: Acute respiratory distress syndrome
RCTs: Randomized controlled trials
CRP: C-reactive protein
SIRS: Systemic inflammatory response syndrome
C3: Complement 3
C4: Complement 4
WBCs: White blood cells
PCT: Procalcitonin
MMP: Mitochondrial membrane potential
ROS: Reactive oxygen species
ATP: Adenosine triphosphate
CYP1A1: Cytochrome P4501A1
UGT2B7: UDP-glucuronosyltransferase 2B7
MRP2: Multidrug resistance protein 2
